# Decrease in anterior cingulate cortex GABA in schizophrenia at early stage

**DOI:** 10.1192/j.eurpsy.2023.1272

**Published:** 2023-07-19

**Authors:** A. Manzhurtsev, S. Nevzorova, M. Ublinskiy, L. Mosina, I. Melnikov, G. Mamedova, V. Ushakov, N. Zakharova, M. Shlyapnikov, D. Andreyuk, T. Akhadov

**Affiliations:** 1 Emanuel Institute of Biochemical Physics of the Russian Academy of Sciences; 2 Clinical and Research Institute of Emergency Pediatric Surgery and Trauma; 3 Moscow State University; 4 National Research Nuclear University MEPhI; 5 Psychiatric Clinical Hospital 1 named N.A. Alekseev; 6 Institute for Advanced Brain Studies, Moscow State University, Moscow, Russian Federation

## Abstract

**Introduction:**

There is evidence that the concentrations of the main inhibitory neurotransmitter (GABA) may be altered in schizophrenia. The purpose of this study is to find the changes in the GABA concentration in the area of anterior and posterior cingulate cortex of patients with early-stage schizophrenia using the spectral-edited magnetic resonance spectroscopy.

**Objectives:**

To measure the cerebral concentrations of the gamma-aminobutyric acid in schizophrenia patients at early stage.

**Methods:**

Thirty-one subject, 18 controls (11m+7f, 29.6±5.7 y.o.) and 13 schizophrenia patients (F20.0, 8m+5f, 27.5±3.1 y.o.). Philips Achieva dStream 3T MRI scanner, standard head coil. The 3D T1w head images and MEGA-PRESS GABA spectra in ACC and PCC areas were acquired with the following parameters: 50x25x25 mm, TR = 2 s, TE = 64 ms, 180-editing pulses applied at 1.9 ppm and 7.6 ppm, NSA = 288 (acq.time ~10 min). GABA spectra were processed in Gannet program. The intensities of the GABA, Glutamate+glutamine (Glx), creatine (Cr) and unsuppressed water signals were acquired. T-test was used in search for between-group differences.

**Results:**

In ACC region, significant reduction of the GABA/Water was observed (by ~15%, p=0.02) as well as a trend to a decrease in GABA/Cr (by ~10%, p=0.07) in schizophrenia. In PCC, no significant GABA/Water or GABA/Cr differences were observed. Glx/Water and Glx/Cr in both areas were also unchanged.

**Image:**

**Figure:**
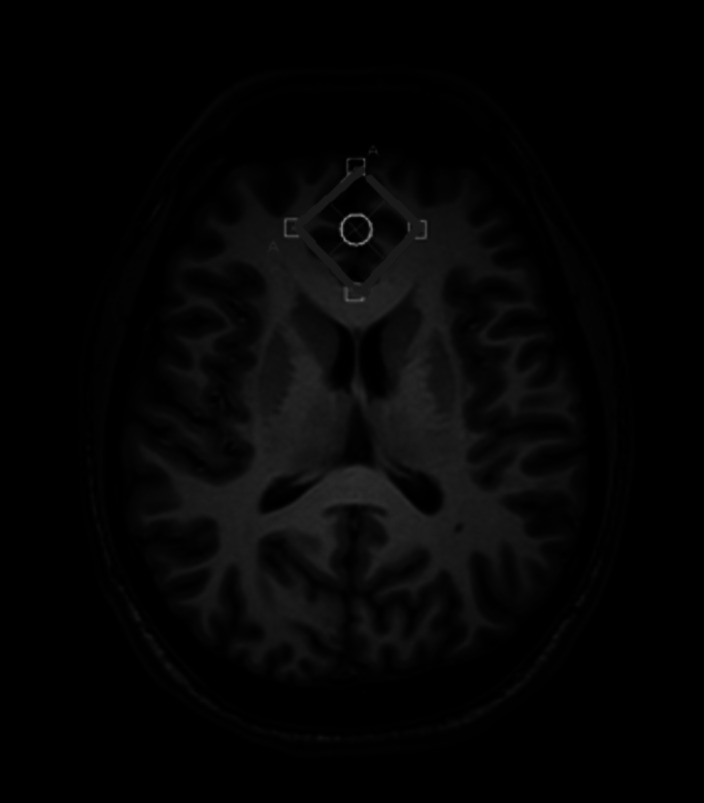

**Image 2:**

**Figure:**
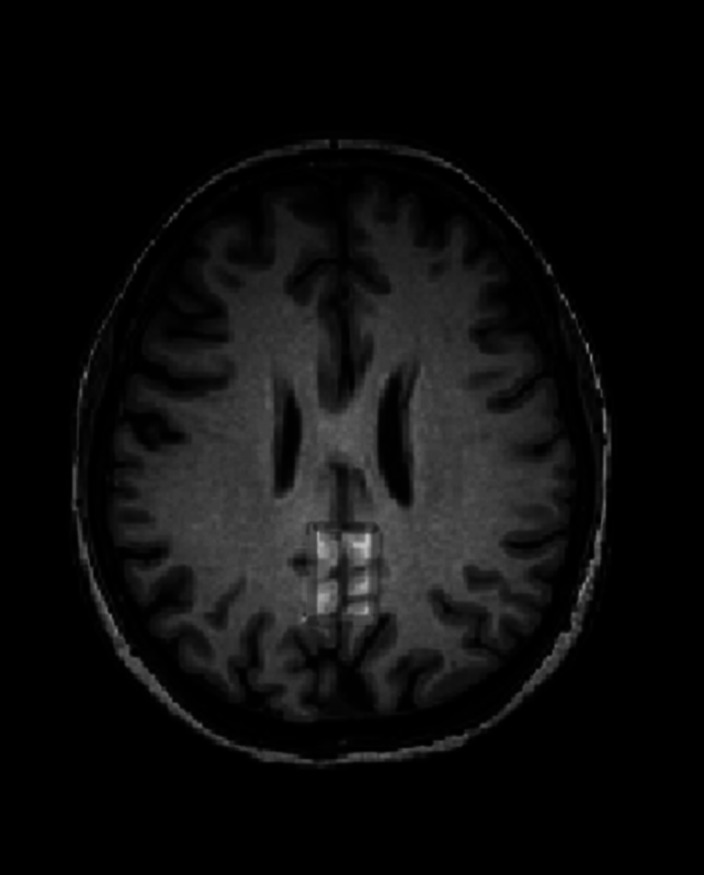

**Conclusions:**

This study provides insight into neurotransmitter alterations at early-stage schizophrenia. The results demonstrate the region-specific changes in the balance of the main neurotransmitters. Since this balance is crucial for the normal cerebral functioning, the results may facilitate better understanding of the dynamics of the pathological process and provide additional information for understanding the biological mechanisms of the schizophrenia development.

**Disclosure of Interest:**

None Declared

